# Changes in depressive symptoms and correlates in HIV+ people at An Hoa Clinic in Ho Chi Minh City, Vietnam

**DOI:** 10.1186/s12888-016-1170-5

**Published:** 2017-01-21

**Authors:** Van-Anh N. Huynh, Kien G. To, Dung Van Do, Quyen G. To, Mai T H Nguyen

**Affiliations:** 1 Faculty of Public Health, University of Medicine and Pharmacy, 217 Hong Bang, District 5, Ho Chi Minh City, Vietnam; 2 Faculty of Health, School of Exercise and Nutrition Sciences, Queensland University of Technology, Victoria Park Road Kelvin Grove, Brisbane, Queensland 4059 Australia; 3 Center for Preventive Medicine in District 6, A14/1 Ba Hom, cu xa Phu Lam D, P13, Quan 6, Tp.HCM, Vietnam

**Keywords:** HIV, Depression, Correlate, ART, Vietnam

## Abstract

**Background:**

Understanding of depression among Vietnamese people living with HIV (PLWH) is limited. This longitudinal study examines changes in depressive symptoms and identifies its correlates among people living with HIV under antiretroviral therapy at An Hoa Clinic.

**Methods:**

People living with HIV ≥18 years and undergoing antiretroviral therapy for ≥3 months were eligible. Those at final AIDS stage, too ill, or illiterate were excluded due to their inability to complete the self-administered questionnaire. One researcher was present in the clinic for a month inviting PLWH to participate. Data were collected from 242 PLWH at baseline (T1) and 234 after three months (T2). Depressive symptoms was measured by the Center for Epidemiologic Studies Depression Scale (CESD). Social relationship was measured using questions created by World Health Organization. Generalized Estimating Equations were used examining changes in depressive symptoms with CESD cut-off <16/≥16 (mild depression) and cut-off <23/≥23 (major depression).

**Results:**

Model 1 (CESD cut-off <16/≥16) showed that participants were not more likely to have depressive symptoms at T2 compared to T1 (OR = 1.15, *p* > 0.05). Those with a co-morbidity were more likely to have depressive symptoms than those without a co-morbidity (OR = 1.76, *p* < 0.05). Those with higher social relationship scores were less likely to have depressive symptoms than those with lower scores (OR = 0.76, *p* < 0.001). Model 2 (CESD cut-off <23/≥23) showed that participants were more likely to have major depressive symptoms at T2 compared to T1 (OR = 1.6, *p* < 0.01) and those with higher social relationship score were less likely to have major depressive symptoms than those with lower scores (OR = 0.73, *p* < 0.001).

**Conclusions:**

People living with HIV were not more likely to have depressive symptoms (<16/≥16) but were more likely to have major depressive symptoms (<23/≥23) at T2 vs. T1. Social relationship was found to be strongly associated with depressive symptoms. Associations between age, individual income status, and co-morbidity with depressive symptoms were not decisive. Gender, ethnicity, education, religion, marriage, household economy, and adherence were not correlates.

## Background

Depression is one of the most common mental health diagnoses among people living with HIV/AIDS (PLWH) [1]. The lifetime prevalence of depression among PLWH was estimated between 22% - 45% [2]. The 12-month prevalence of major depression was 22% in a nationally representative cohort of PLWH receiving care in the U.S. [3]. Although the rate among PLWH may or may not be different from other adults in the same community, it seemed to be lower for the general population [1, 4, 5]. The prevalence rate for major depression was 6.6% among adults participating in a U.S. representative household survey [6].

Studies on changes in depressive symptoms found mixed results. Although one study found that the mood was stable regardless of illness progression [7], another study showed that depression decreased over a two-year period after receiving ART [8]. Others showed that depression did not change until the period of 12–18 months before AIDS diagnosis when depression started increasing significantly [9–11].

Depression was found to be associated with HIV progression and mortality among PLWH [12–15]. In addition, PLWH with depression were more likely to have poor medication adherence [16–19]. Those with depression and poor adherence had a mortality risk of six times higher than those without depression [20]. Effective treatment of depression also improved adherence to antiretroviral therapy (ART) [21]. Other correlates of depression included age, gender, substance abuse, perceptions of HIV related stigma, social support and social isolation [1, 22–27].

Vietnam is a Southeast Asian country with over 90 million people. It is estimated that 197,335 people were living with HIV/AIDS in 2011 and 263,317 in 2015 [28]. Because HIV is usually linked to drug use and commercial sex workers [29, 30], which are illegal and culturally unacceptable in Vietnam, PLWH are likely to experience mental health problems, particularly depression, due to stigma and discrimination [31]. Social support and access to mental health care for PLWH are also insufficient in Vietnam [32–34].

Understanding about depression among PLWH in Vietnam is limited. In our literature review, we found only two cross-sectional studies on depression among PLWH in Vietnam. One showed that depression prevalence among HIV-infected men over a month was 18.7%, which seems to be much higher than the general population of Vietnamese men [35]. The second article found an inverse association between ART adherence and depression [36].

This study, therefore, aims to address this gap by (1) examining changes in depression symptoms among PLWH at An Hoa clinic in Ho Chi Minh city (HCMc), Vietnam; and (2) identifying correlates of depression. Findings from this study will improve knowledge about depression among PLWH in Vietnam and help design effective interventions.

## Methods

### Study design and population

This longitudinal study was conducted from November 2012 to April 2013 at An Hoa Clinic of Center for Preventive Medicine in District 6, HCMC, Vietnam. PLWH who were 18 years or older; undergoing ART for ≥3 months; and agreed to participate were eligible. Those at final AIDS stage, too ill, or illiterate were excluded due to their inability to complete the self-administered questionnaire.

One researcher stayed at the clinic during operating hours for one month and approached PLWH who visited the clinic for services, in order to screen them for eligibility and invite them to participate in the study. PLWH were given information about the study and written consent forms were collected if they agreed to participate. During the month, the researcher approached 255 of 1260 PLWH registered in the clinic. Of these participants, two were illiterate, one was at end stage of AIDS; and two were not receiving ART. Of 250 PLWH who agreed to participate, two subsequently died, four were incarcerated, and two later withdrew at the first administration. Therefore, the final sample size at baseline was 242. Because our resources were limited and two previous studies showed a period of three month follow-up appropriate to see changes in depressive symptoms [37, 38], data were collected at baseline (T1) and after three months (T2) from T1. The study was approved by the Research Ethics Committee, University of Medicine and Pharmacy, Ho Chi Minh City and the Clinic Administrative Board.

### Measurement

Self-administered questionnaires were provided to the participants when they visited the clinic. Record review and pill counts were used along with self-reports to determine adherence.

### Dependent variable

Depression was measured by the Center for Epidemiologic Studies Depression Scale (CESD). CESD was created in 1977 and widely used to measure depression in communities [39, 40]. Particularly, it was validated among Vietnamese [40]. CESD measures nine domains of depression defined by the American Psychiatric Association [41] including Sadness, Loss of interest, Appetite, Sleep, Thinking/concentration, Guilt, Tired, Movement, and Suicidal ideation. It is comprised of 20 items, each of which is rated as 0 = “Rarely or none of the time (<1 day)”, 1 = “Some or a little of the time (1–2 days)”, 2 =“Occasionally or a moderate amount of time (3–4 days)”, and 3 = “Most or all of the time (5–7 days)”. Four positive items have the scores reversed. As such, the total score ranges from 0 to 60. Two categorical variables were created as “possible depression” or “mild depressive symptoms” where the cut-off point is ≥/<16; and “probable depression” or “major depression” where the cut-off point is ≥/<23 [39]. However, we only used the terms “mild depression” and “major depression” for consistency. Cronbach’s alpha for T1 and T2 were 0.87 and 0.89 which showed strong internal consistency reliability.

### Independent variables

#### Social relationships among PLWH

##### Social Relationships

Were measured by four questions created by the World Health Organization (WHO-QOL BREF) [42]. The first one is: “To what extent do you feel accepted by the people you know?” and five responses are “Not at all”, “A little”, “Moderately”, “Mostly”, and “Completely”. The other three are “How satisfied are you with your personal relationships?”, “How satisfied are you with your sex life?”, and “How satisfied are you with the support you get from your friends?” and the responses are “Very dissatisfied”, “Dissatisfied”, “Neither satisfied nor dissatisfied”, “Satisfied”, and “Very satisfied”. The score for each question is from one (negative) to five (positive). The total score, therefore, ranges from 4 to 20. Cronbach’s alpha for T1 and T2 were 0.66 and 0.76 which showed good internal consistency reliability.

#### Medication adherence

A definition of medication adherence by the Vietnam Ministry of Health was modified to measure adherence [43]. First, two yes-no questions were asked: (i) “During the last month, have you ever given your medication to others?”; and (ii) “During the last month, have you ever forgotten to take your medication?” Second, although self-reports of adherence are fairly accurate [44], returned pills were counted to cross-validate. A mismatch between self-reports and pill counts resulted in an answer of “Yes” for the question (ii). Third, the appointment date was checked to see whether the participant was late, i.e., did not re-visit the clinic at the appointed date or within the following day. An answer of “Yes” to any of these resulted in coding of poor adherence.

Other independent variables including co-morbidity, age, gender, ethnicity, education, marital status, religion, individual income status, and household economy were self-reported. Co-morbidity was “Yes” if a respondent had at least one of these diseases including tuberculosis, hepatitis B or C, cardiovascular disease, or kidney diseases. Age groups were “18-30 years”, “31-40 years”, and “41-59 years”. Gender was “Female” or “Male”. Ethnicity was “Kinh people” (main ethnic group in Vietnam) or “Others”. Participants’ levels of education were grouped into “Below Grade 10” or “≥Grade 10”. Responses for marital status were grouped into “Living with a spouse/partner” or “Others”. Responses for having a religion were “Yes” or “No”. Individual income status was responded as “Regular”, “Irregular”, or “No income”. Household economy was categorized as “Economically disadvantaged” or “Not economically disadvantaged”.

### Data analysis

Data were entered using Epidata 3.1 software. Analyses were performed with the use of SAS software, v9.4. Frequencies and percentages were generated for categorical variables. Means and standard deviations were generated for continuous variables. Chi-square test was used to see whether there were differences in demographic characteristics of the samples at two time points. Two models with CESD cut-off of 16 “mild depression” (model 1) and 23 “major depression” (model 2) were run. Although the first cut-off (<16/≥16) was traditional and used in most research with CES-D, the second cut-off (<23/≥23) was also used because somatic depressive symptoms on CES-D can overlap with HIV-related disease symptoms (e.g., poor appetite) and inflate CES-D scores [45]. Potential correlates were included in these two models and odd ratios were reported for each correlates. Tukey-Kramer adjustment was applied when conducting multiple comparison for two variables: age and individual income status. All *p*-values are two tailed and *p*-values of <0.05 were considered statistically significant.

Generalized Estimating Equations (GEE) were used to examine changes in depressive symptoms. GEE is a suggested method to analyze correlated longitudinal data [46]. It provides consistent and robust estimation of parameters’ standard errors and can be applied to different types of outcome variables (e.g., continuous, dichotomous, and count). GEE can account for unequal intervals among waves and for missing data in longitudinal studies.

## Results

### Characteristics of the sample

At baseline, the average age of participants was 33 years, ranging from 18 to 59 years; about 62% were from 31–40 years (Table 1). Female made up 26.86% of the sample. A majority were “Kinh people” (85.54%); did not go to senior high school (78.10%); and had a religion (88.43%). About half were living with a spouse (52.89%); and had a co-morbidity (51.65%). A quarter (24.79%) had no income. Over one-third (40.08%) lived in economically disadvantaged household. About 70.66% adhered to treatment. The mean of the social relationship score was 12.33. Of 242 participants, 61.98% (150 participants) had depression and 34.71% (84 participants) had major depression. CESD mean score was 19.91.

**Table 1:** Characteristics of the samples

	T1 (*n* = 242)		T2 (*n* = 234)	
	N	Mean (SD) or %	N	Mean (SD) or %
Age				
18–30	67	27.69%	67	28.63%
31–40	151	62.40%	143	61.11%
41-59	24	9.92%	24	10.26%
Gender (Female)	65	26.86%	64	27.35%
Ethnicity (Kinh people)	207	85.54%	200	85.47%
Education (Below Grade 10)	189	78.10%	183	78.21%
Religion (Yes)	214	88.43%	209	89.32%
Marital status (Live with spouse)	128	52.89%	123	52.56%
Individual income status				
Regular	106	43.80%	108	46.15%
Irregular	76	31.40%	69	29.49%
No income	60	24.79%	57	24.36%
Household economy (economically disadvantaged)	97	40.08%	86	36.75%
Co-morbidity (Yes)	125	51.65%	125	53.42%
Adherence (Yes)	171	70.66%	194	82.91%
Social relationship score	242	12.33 (2.78)	234	12.33 (2.91)
CESD mean score (SD)	242	19.91 (10.33)	234	20.56 (10.23)
CESD cut-off (≥16)				
Yes	150	61.98%	148	63.25%
No	92	38.02%	86	36.75%
CESD cut-off (≥23)				
Yes	84	34.71%	99	42.31%
No	158	65.29%	135	57.69%

*CESD* the Center for Epidemiologic Studies Depression scale

*SD* Standard Deviation

The number of participants at T1and T2 was, respectively, 242 and 234. Chi-square test showed that differences in demographic characteristics at the two time points were not significant (*p* = 0.904 for gender, *p* = 0.959 for age groups, *p* = 0.983 for ethnicity, *p* = 0.978 for education level, and *p* = 0.759 for religion).

### Changes in depressive symptoms and correlates

Model 1 with CESD cut-off of 16 showed that participants were not more likely to have depressive symptoms at T2 compared to T1 (OR = 1.15, *p* > 0.05). Model 2 with CESD cut-off of 23 showed that odds of having major depression at T2 compared to T1 was 1.6 (*p* < 0.01) (Table 2).

**Table 2:** GEE for binomial models with CESD cut-off of 16 and 23

	Model 1 - CESD (<16/≥16)		Model 2 - CESD (<23/≥23)	
	OR	95% CI	OR	95% CI
Time 2 vs. Time 1	1.15	(0.86, 1.53)	1.60	(1.13, 2.24)**
Age				
(18–30) vs. (31–40)	0.87	(0.43, 1.72)	0.63	(0.32, 1.24)
(18–30) vs. (41–59)	0.72	(0.24, 2.22)	0.38	(0.14, 1.06)
(31–40) vs. (41–59)	0.83	(0.30, 2.33)	0.60	(0.28, 1.30)
Female vs. Male	1.06	(0.60, 1.88)	1.21	(0.70, 2.11)
Kinh people vs. others	0.72	(0.33, 1.54)	0.69	(0.38, 1.28)
Below vs. Above grade 10	1.32	(0.76, 2.26)	1.11	(0.62, 1.99)
Religion (Yes vs. No)	0.80	(0.38, 1.69)	1.01	(0.46, 2.19)
Live with spouse vs. not	1.02	(0.64, 1.64)	1.10	(0.69, 1.75)
Individual income status				
Regular vs. irregular	1.08	(0.58, 2.04)	0.63	(0.34, 1.20)
Regular vs. no income	0.84	(0.41, 1.72)	0.57	(0.29, 1.10)
Irregular vs. no income	0.78	(0.35, 1.72)	0.89	(0.46, 1.72)
Household economy (Not economically disadvantaged vs. disadvantaged)	0.69	(0.45, 1.04)	0.90	(0.55, 1.48)
Co-morbidity (Yes vs. No)	1.76	(1.15, 2.70)*	1.23	(0.77, 1.98)
Social relationship (one unit difference)	0.76	(0.69, 0.82)***	0.73	(0.66, 0.81)***
Adherence (Yes vs. No)	0.73	(0.47, 1.16)	0.69	(0.44, 1.09)

Note: the models included all covariates

Probability of having depression was modeled *p < 0.05;**p < 0.01; ***p < 0.001

*OR* odds ratio, *CESD* the Center for Epidemiologic Studies Depression scale, *GEE* Generalized Estimating Equations

Model 1 found two correlates with depression, co-morbidity and social relationship. Those with a co-morbidity were more likely to have depressive symptoms than those without a co-morbidity (OR = 1.76, *p* < 0.05). Those with higher social relationship score were less likely to have depressive symptoms than those with lower scores (OR = 0.76, *p* < 0.001). Model 2 found that only social relationship was associated with depression. Those with higher social relationship score were less likely to have major depression than those with lower scores (OR = 0.73, *p* < 0.001). In both models, depression was not significantly associated with other variables such as age, gender, and medication adherence (*p* > 0.05).

## Discussion

The findings showed that PLWH were not more likely to have depressive symptoms (CESD ≥ 16) at T2 compared to baseline (model 1) but were more likely to have major depression (CESD ≥ 23) at T2 compared to baseline (model 2). This may suggest that progression from not having depression (CESD < 16) to having depression was not as significant as the progression from having mild to major depression. One possible explanation may be because those who already had mild depression were more likely to live in an unfavorable environment where stigma, lack of mental health services, and insufficient support were more popular than those without depression (i.e., CESD < 16). As environmental effects accumulated, depression symptoms increased. However, this is speculative since data on environment, especially social environment, were not collected. In addition, although it is possible that the effect of previous exposure to the questionnaire may influence responses at T2 (e.g., PLWH responded with more depression at T2 expecting to receive more support), it may not be plausible because differences in depression was not significant in model 1.

Although reliable population comparisons are not available, high prevalence of major depression (34.31%) in the study sample likely indicates that interventions are needed to prevent and control depression among PLWH.

Despite that women were found more likely than men to suffer depression in the general population [47], the association was less consistent among PLWH [1, 48–50]. In this study, women seemed to have more depression although the association was insignificant. It is worth noting that women made up only 26% of the sample, and so the statistical power to detect differences between the sexes may be limited.

Medication adherence was not associated with depression in this study. This is not consistent with other studies which found that depression was associated with poorer adherence [16, 17, 36]. This may be due to PLWH’s reactivity, i.e., trying to improve adherence, as they may think that they were under a close supervision when participating in the study. However, it is also worth noting that this study used a stricter definition of adherence by which remembering to take medication was not the only criterion. PLWH needed to revisit the clinic on the appointment date or the next day at the latest. As such, the finding may not be comparable to other studies.

Having a co-morbidity seems to be associated with greater likelihood of depression. Although the association was only significant in model 1 (CESD <16/≥16), the direction of the effect was the same in model 2 (ORs > 1). The effect of co-morbidity on depression is plausible due to the impact of deteriorating physical and mental health and the stress of cost and side-effects related to treatment medication for co-morbidities.

Although depression can be effectively treated among PLWH [51, 52], interventions should focus not only on treating depression but also treating other co-morbidities. In addition, the finding that social relationship was the strongest correlate with depression in this study emphasized the importance of addressing psychosocial needs of PLWH. Improving social relationship should be included as an essential strategy in future interventions.

Although our study had an appropriate design, used validated measurement tools, and had an adequate sample size, it has some limitations. First, this study was conducted in only one clinic so generalizability was limited. Second, depression were self-reported so they may not be as accurate as clinical diagnosis. However, as the same self-reported tool was used at both T1 and T2, the effect of this bias would be limited. Third, although the instruments were validated, recall bias is possible in self-reported data. Fourth, as depression treatment was not controlled for, it is possible that some received depression treatment elsewhere during the three-month study period. However, the chance was minimal because depression care was very limited in Vietnam [34]. Mental health services, even in big cities such as Hanoi and Ho Chi Minh city, do not focus on depression but mainly on schizophrenia, bipolar disorder, and epilepsy [34]. Finally, since this is an observational study without a comparison group, many factors which could affect depression could not be fully controlled.

## Conclusion

PLWH were not more likely to have depression (<16/≥16) at T2 compared with baseline but more likely to have major depression (<23/≥23) at T2 compared with baseline. Social relationship was found to be strongly associated with depression. Associations between age, individual income status, and co-morbidity were not decisive. Gender, ethnicity, education, religion, marriage, household economy, and adherence were not correlates.

Our study was a response to a call for more studies on HIV and depression in Asian countries [53]. It contributes to understanding of depression among PLWH receiving ART in Asia, and particularly in Vietnam. Our findings emphasize the need for interventions addressing mental health among PLWH in Vietnam. This study can be useful in designing and building effective interventions to prevent and control depression. Future research should include larger and more diverse samples to increase generalizability; control effects associated with other factors such as previous exposure to the questionnaire; improve depression diagnosis by clinical assessment; and evaluate severity of co-morbidities to increase the strength of conclusions.
